# Nanomaterials as Novel Cardiovascular Theranostics

**DOI:** 10.3390/pharmaceutics13030348

**Published:** 2021-03-07

**Authors:** Rajasekharreddy Pala, Subhaswaraj Pattnaik, Siddhardha Busi, Surya M. Nauli

**Affiliations:** 1 Department of Biomedical and Pharmaceutical Sciences, Harry and Diane Rinker Health Science Campus, Chapman University, Irvine, CA 92618, USA; 2 Department of Medicine, University of California Irvine, Irvine, CA 92868, USA; 3 Department of Microbiology, School of Life Sciences, Pondicherry University, Puducherry 605014, India; pattnaiksslifesc26590@gmail.com (S.P.); siddhardha.mib@pondiuni.edu.in (S.B.)

**Keywords:** diagnosis, bioimaging, therapeutics, nanotheranostics, CVDs

## Abstract

Cardiovascular diseases (CVDs) are a group of conditions associated with heart and blood vessels and are considered the leading cause of death globally. Coronary heart disease, atherosclerosis, myocardial infarction represents the CVDs. Since CVDs are associated with a series of pathophysiological conditions with an alarming mortality and morbidity rate, early diagnosis and appropriate therapeutic approaches are critical for saving patients’ lives. Conventionally, diagnostic tools are employed to detect disease conditions, whereas therapeutic drug candidates are administered to mitigate diseases. However, the advent of nanotechnological platforms has revolutionized the current understanding of pathophysiology and therapeutic measures. The concept of combinatorial therapy using both diagnosis and therapeutics through a single platform is known as theranostics. Nano-based theranostics are widely used in cancer detection and treatment, as evident from pre-clinical and clinical studies. Nanotheranostics have gained considerable attention for the efficient management of CVDs. The differential physicochemical properties of engineered nanoparticles have been exploited for early diagnosis and therapy of atherosclerosis, myocardial infarction and aneurysms. Herein, we provided the information on the evolution of nano-based theranostics to detect and treat CVDs such as atherosclerosis, myocardial infarction, and angiogenesis. The review also aims to provide novel avenues on how nanotherapeutics’ trending concept could transform our conventional diagnostic and therapeutic tools in the near future.

## 1. Introduction

Since the discovery of antibiotics, a significant development in the biomedical and healthcare sectors’ technological interventions has revolutionized the current understanding of disease diagnosis and therapy. In the last few decades, the global healthcare network’s advancement has also provided a platform to protect and maintain human beings’ health profiles [1]. However, the world has also witnessed a similar but exponential incidence of a series of infectious and non-communicable diseases (NCDs). Like infectious diseases, NCDs also gained considerable attention in the last few decades due to the high mortality and morbidity rate. The evolution of cardiovascular diseases (CVDs), respiratory ailments, diabetes, and cancer are the most prominent diseases and disorders that account for most health consequences and mortality rates in the 21st century. The emergence of NCDs becomes a public health issue owing to its global impact on human health, economy and society. NCDs could account for 70% of global death by 2025, as estimated by the World Health Organization (WHO), with a significant implication on developing countries. Among them, approximately 84% of global death will occur due to the increased incidence of CVDs (48%), cancer (21%), chronic respiratory ailments (12%) and diabetes (3%) by 2025 [2]. The world is currently witnessing the high risk of comorbidities in chronic medical conditions, particularly in the patients with CVDs co-occurring with the COVID-19 pandemic, thus providing an alarming risk to the healthcare settings.

CVDs include a collection of several health conditions, including atherosclerosis, cardiomyopathy, arrhythmia, myocardial infarction, coronary artery disease, aneurysm and hypertension. Since CVDs infer the assemblage of several diseased conditions and disorders, the occurrence of CVDs become a severe global trend. The emergence of CVDs poses serious health complications resulting in severe implications on any given country’s economy. As per the data available, approximately 17.7 million people died across the globe in 2015 due to the complications associated with CVDs. In this context, it is estimated that by 2030, the mortality due to CVDs could rise to 22 million. In this context, the advancement in the technological perspectives could provide an array of therapeutic alternatives to minimize the nuisances of chronic diseases and disorders associated with CVDs. However, the co-occurrence of CVDs and chronic infectious ailments poses a severe challenge to the current therapeutic strategies. Immunotherapy, chemotherapy, and other conventional approaches are considered as the frontline warriors to fight against CVDs. The concept of host-directed therapies also gained considerable attention in the last few years for their ability to fight against NCDs, particularly CVDs [3]. Though significant work is going on to develop and/or curate the conventional therapies to improve the healthcare settings in an emergency, these strategies fall short due to one or other limitations [4]. In this regard, it is imperative to look for an effective, alternative and exact method for timely diagnosis of CVDs followed by effective treatment procedures. The genome-based approaches are also found to be influential in disease diagnosis and therapy [5].

### 1.1. Theranostics: An Overview

For the management of any chronic diseases and/or disorders, the foremost important thing is the early diagnosis of disease progression and based upon the diagnostic information, implementing the proper therapeutic measures. In this regard, approximately two decades ago, a combinatorial modality involving both diagnosis and therapeutics came into the limelight. The integrated concept of ‘theranostic’ was derived from therapy and diagnostics. The term “theranostics,” coined by John Funkhouser in 2002, refers to an assemblage of diagnostic tools and appropriate therapeutic measures under a single platform [6,7]. The theranostic tools have provided a customized platform for target-guided therapy of chronic disease conditions utilizing the diagnostic and therapeutic modalities in a single platform. The integrated theranostic approach provided a new dimension to overcome the limitations of differential diagnosis and treatment procedures [7,8]. The evolution of theranostic strategies centered around the target-specific diagnostics and therapies and critically distinguished the healthy or non-targeted sites from any lethal effect [9]. The notable advantages of theranostic approach are low toxicity, selectivity, target-specificity, and tunability [10]. Several internal markers such as variations in pH, compliance of redox reactions, cellular enzymatic level, and response from genetic materials are influential in theranostic functional modalities [11].

The advent of theranostics has provided a selective platform for transforming conventional therapeutic medicines to personalized and particular treatments, suggesting a more holistic strategy to counteract health consequences of chronic health disease’ [12]. The concept of theranostics critically expressed its candidature as next-generation personalized medicines. It allows a series of sequential steps such as early detection and diagnosis of disease, disease prognosis, therapy selection, and monitoring the therapeutic efficacy with high precision selectivity [13]. Since the limitations such as radiation-associated health hazards and inference of allergic reactions are associated with radionuclides during chemotherapy and nuclear medicines, theranostics could guide the development of next-generation nuclear drugs [14]. The specificity with which the theranostic modalities integrate the diagnostics and therapeutics through a common platform could lead the scientific community as a mainstream arsenal against chronic diseases and other health ailments [15]. The theranostic modalities provide a non-invasive platform to diagnose disease conditions and targeted therapy. This concept received remarkable recognition in the fight against infectious diseases, inflammatory disorders, and cancer therapy.

### 1.2. Nanotheranostics: An Emerging Trend

**Figure 1:**
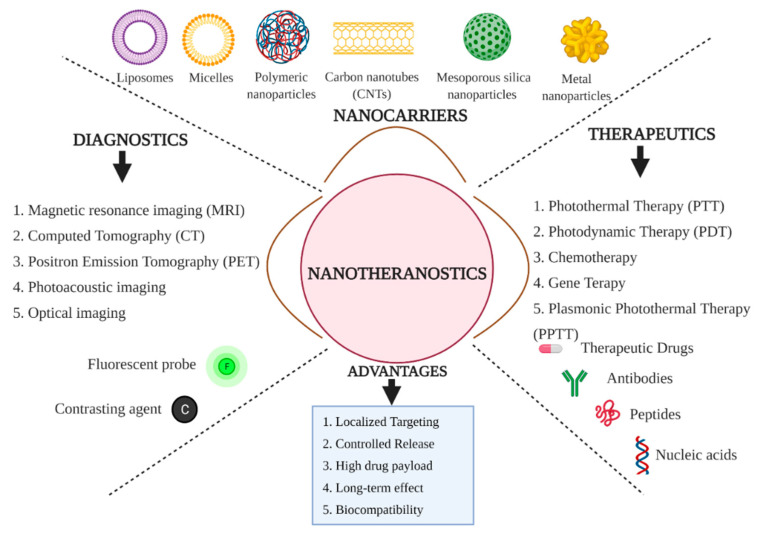
Schematic overview of the concept of nanotheranostics which infers the use of engineered nanomaterials for both diagnostics and therapeutic modules through a single platform.

Nanotechnology in biomedicine and healthcare settings has resulted in the paradigm shift in understanding the disease prognosis and efficient therapeutic strategies to overcome chronic health consequences. The nano-based platforms provided a multifaceted platform for rapid and early diagnosis of disease progression. Furthermore, they exhibited therapeutic applications, including spatial targeting of therapeutic drug moieties and tracking of therapeutic response from the cellular system using different therapeutic modules such as gene therapy, chemotherapy, photodynamic therapy and photothermal therapy with differential specificity in terms of therapeutic efficacy [16,17,18] (Figure 1). The emerging trend of nanotechnology in biomedical and healthcare settings has revolutionized the scientific upliftment of disease diagnosis and therapy with widespread potential. The nanotechnology platforms could be highly considered theranostic modules for effective diagnosis and treatment due to the physicochemical properties such as high surface-to-volume ratio, tunability, ease of chemical characterization, and surface properties modification [19]. The concept of ‘nanotheranostics’ is an advanced version of theranostic modalities with nanomaterials of differential class and physicochemical properties for disease diagnosis and optimized therapies [20]. The nanotheranostics represent the most advanced technological intervention with multifaceted functional attributes such as multimodal imaging, drug targeting and improved synergistic therapeutics [21].

The engineered nanomaterials of differential classes such as magnetic nanoparticles, carbon-based nanomaterials, silica-based nanomaterials, metal-based nanoparticles, and polymeric nanoparticles exhibited bimodal applications in terms of both diagnostics and therapeutic perspectives. In particular, the light-emitting and light-responsive nanomaterials, such as metal-based nanoparticles with high plasmon resonance (HPR), semiconductor quantum dots (QDs), organic and polymeric nanomaterials, gained considerable attention as cost-effective and efficient theranostic agents [22]. The unique structural dimension, unique physicochemical properties, ease of functionalization, improved biocompatibility, and, most importantly, the broad range of one-photon properties of carbon-based nanomaterials are considered landmarks characteristics for biomedical applications. Carbon-based nanomaterials such as carbon nanotubes (CNTs), QDs, graphene oxide (GO) are being exploited as bimodal applications in the field of diagnostics and therapeutics [23]. Apart from carbon-based nanomaterials, polymeric nanoparticles and their combination with different nanomaterials have given a new dimension to nanomaterials mediated theranostics for multifaceted functional attributes. The exploitation of combinatorial nanomaterials found to be influential in disease diagnosis and management [24]. The chemistry associated with the conjugated polymeric nanoparticles strictly allows multimodal imaging of deeply situated tissues with spatial resolution when used as contrasting agents in near-infrared imaging (NIR imaging), two-photon imaging, and photoacoustic imaging. Thus, conjugated polymeric nanomaterials were found to be involved in the early diagnosis of disease. Besides, these conjugated nanomaterials are also observed to influence therapeutic modalities such as photothermal therapy (PTT) and photodynamic therapy (PDT), which are considered to be influential in non-invasive therapeutics of cancer as clinically approved earlier and hence could be instrumental in CVDs management [25,26].

The highly defined and unique physicochemical properties of nanomaterials are being exploited as prolific contrasting agents, which proves to be instrumental in the early detection and diagnosis of disease prognosis. Hence, nanomaterials are considered effective bioimaging modalities. Nanomaterials are being used as effective contrasting agents for various conventional diagnostic tools such as optical imaging, ultrasonography, magnetic resonance imaging (MRI), positron emission tomography (PET), photoacoustic tomography and computed tomography (CT). Thus, the role of nanomaterials as bioimaging tools has guided the scientific community towards bioimaging-guided therapeutics against NCDs [10].

The ease of synthesis, unique physicochemical characteristics, and bimodal applications associated with the nanomaterials provided a unique platform for detecting and redefining the therapeutic measures for various chronic healthcare conditions, including neurodegenerative diseases Alzheimer’s disease and Parkinson’s disease [15]. The nanotheranostic tools received potential consideration for cancer therapy as the modalities allowed to detect and diagnose cancer progression through bioimaging techniques and provide a novel drug delivery system for systemic and controlled delivery of therapeutic agents at the target sites bypassing several cellular barriers [27]. The emergence of nanotheranostic modalities has given a new dimension to the concept of personalized nanomedicines for efficient management of cancer, inflammatory diseases, CVDs, neurodegenerative diseases, diabetes and other chronic health ailments [28,29]. As the nanotheranostic tools involve differentially exclusive nanomaterials, the functional attributes also vary greatly as per the requirement. For example, theranostic nanomedicine could exploit various inorganic nanoparticles as effective contrasting agents for multimodal imaging. Similarly, the nanotheranostic platforms can also encapsulate a number of therapeutic drug moieties and contrasting agents, thus from a single platform, both the bioimaging and therapeutic attributes could be considered. In this context, recently spatially designed multifunctional co-loaded magnetic nanocapsules (MNCPs) were developed, which could be used not only for enhanced bioimaging modalities but also could be instrumental in targeted photodynamic therapy [30]. In a recently reported research work, it was evident from the spatially designed and fabricated phosphatidylcholine/cholesterol-based liposome modified by sodium cholate hydrate could be considered as a bimodal platform for diagnostics as well as therapeutics [31]. The nanostructures’ tunability allows them to load various therapeutic drug moieties, target-specific ligand molecules, and specific antibodies for target-oriented functional attributes [32].

## 2. Recent Trends in Therapies Associated with Cardiovascular Diseases

**Figure 2:**
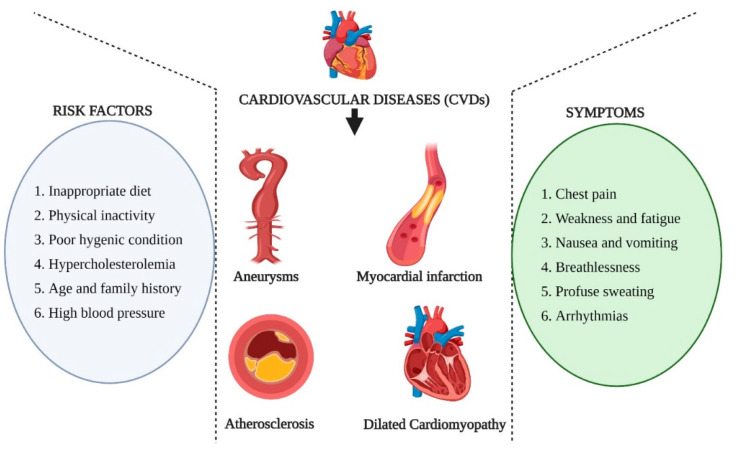
Schematic representation of different types of cardiovascular diseases (CVDs), the risk factors associated with the occurrence of CVDs and the common pathophysiological symptoms involved in CVDs.

Among the notable non-communicable diseases in recent decades, CVDs emerged as the most life-threatening disease condition along with cancer in both developed and developing countries. CVDs are pathophysiological conditions of the cardiovascular system, including the blockage of blood vessels, irregular rhythmic function of heart muscles (arrhythmias), myocardial infractions, dilated cardiomyopathy, etc. CVDs are estimated to contribute to the highest percentage of mortalities by the year 2025 among the NCDs. As per the records received in the year 2017, CVDs are responsible for 17.7 million deaths worldwide, which accounts for approximately 1/3rd of the total global deaths suggesting the severity of CVDs [33]. When the CVDs are co-occurring with other physiological ailments, the situation becomes even worse. One of the exciting aspects of CVDs is the high rate of mortalities observed in low and middle-income countries, thus possess potential socio-economic hazards [34]. Several risk factors such as physical inactivity, the occurrence of other pathophysiological conditions, poor hygienic conditions, inappropriate diet systems are highly responsible for aggravating the consequences of CVDs. Hence, the advent of CVDs infers a more significant challenge to the scientific community to develop novel strategies to overcome the health hazards and preservation of humankind from such health consequences (Figure 2).

The conventional therapeutic strategy for vascular diseases includes administering small drug molecules through oral, parenteral, intravenous, and intra-arterial routes. However, systemic side effects, rapid drug clearance, drug instability, and non-targeted localization limits the applications of drug molecules. Apart from administering drug molecules, invasive procedures such as angioplasty, atherectomy, stenting, bypass grafting are commonly employed to treat vascular diseases. However, the lack of bioimaging modalities limits their usefulness. In this context, alternative, non-invasive, target-oriented, and bioimaging assisted modalities could be considered to manage vascular diseases [35].

### Nanoparticles as Theranostic Agents for Cardiovascular Diseases

Since the advent of nanotechnological concepts and their intervention into biological issues, a long-range of spatially designed nanomaterials are being considered for widespread applications in various fields. The emergence of nanobiotechnology has specifically revolutionized the biomedical sectors and characteristically improve the current understanding of various pathophysiological conditions and their guided therapeutic measures. A number of factors such as high surface plasmon resonance (SPR), high surface to volume ratio, tunability, biocompatibility, optical and magnetic properties are responsible for their influence in disease diagnosis and as localized therapeutics [36]. Inorganic nanoparticles (metal and metal oxide-based nanoparticles), organic nanoparticles (Polymer-based nanoparticles), carbon-based and lipid-based nanomaterials exhibited promising attributes as multifunctional theranostic agents. The concept of nanotheranostics has gained recent recognition to implement the management of CVDs and neurological disorders after being highly affirmed in its recognition in cancer therapeutics [37].

## 3. Nanotheranostics for Atherosclerosis

### 3.1. Atherosclerosis: An Overview

Atherosclerosis refers to the pathophysiological conditions where the medium and large arteries are significantly affected due to spatial deposition of various materials such as lipoproteins (particularly cholesterol), immune cells (circulating monocytes), proinflammatory factors (i.e., macrophages and T-cells), degraded extracellular matrix components and necrotic products. Atherosclerosis is considered to be the primary cause of chronic CVDs, including coronary heart disease, cerebrovascular disease, and peripheral arterial disease [38,39,40]. Atherosclerosis is characterized by a series of physiological events, including endothelial dysfunction, inflammatory responses, cell proliferation, lipoprotein deposition, vascular remodeling, and finally, plaque formation [41]. The pathophysiological conditions associated with atherosclerosis are highly influenced by the macrophages as their migration, activation, infiltration and proliferation characteristically promote the formation of inflammated atherosclerotic plaques. The atherosclerotic plaques are composed of lipids (mainly cholesterol followed by phospholipids), inflammatory cells, smooth muscle cells, connective tissue (including collagen and elastic fibers), thrombi and calcium deposits [42]. The inflammation is due to the secretion of several proteases and tissue factors under macrophages’ influence. Hence, macrophage-targeted therapeutics are considered to be promising in minimizing the inflammatory responses and the degradation of atherosclerotic plaques [39]. The design and development of theranostic nanomaterials for the management of atherosclerosis depend upon several physiological markers. One of the exciting biomarkers to target atherosclerotic plaques is fibrin, which characteristically facilitates sensitive detection during plaque formation. In addition to fibrin, α_v_β_3_ integrin is also considered a unique biomarker as it is associated with the process of angiogenesis and transiently expressed when the endothelial layer remains physiologically intact. Hence, the therapeutic measures centered around targeting these important physiological markers when spatially designed nanomaterials are being considered as theranostic materials [43]. Looking into the conventional therapeutics for atherosclerosis, it was observed that drug candidates targeting atherosclerosis promoting factors such as hypertension and dyslipidemias are regularly being considered. In addition, the importance of inflammatory responses during atherosclerotic plaque formation, anti-inflammatory drugs are also being administered to treat atherosclerosis [38]. Additionally, the conventional therapeutics also include FDA-approved statins i.e., β-Hydroxy β-methylglutaryl-CoA (HMG-CoA) reductase inhibitors), fibrates, nicotinic acid and ezetimibe [44].

### 3.2. Nano-Based Platforms for Diagnosis and Treatment of Atherosclerosis

**Figure 3:**
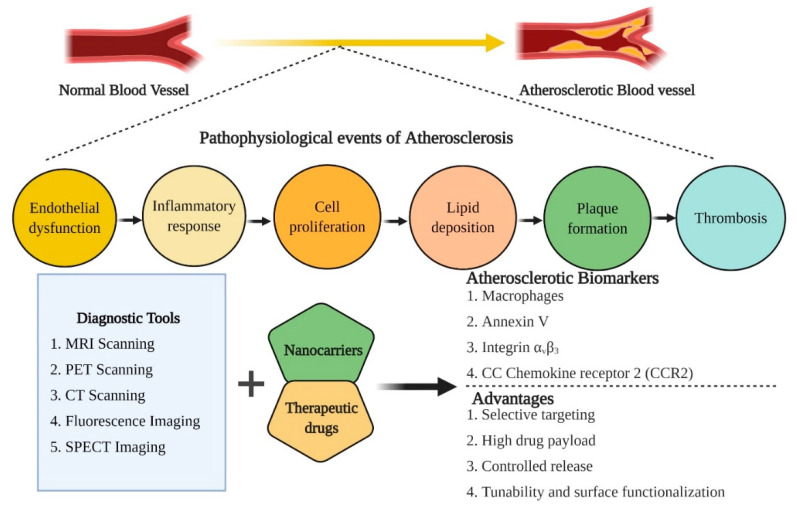
An overview of the pathophysiological events associated with the induction of atherosclerosis, therapeutic biomarkers for diagnosis and treatment of atherosclerosis and the advantages associated with nano-based theranostic tools for the management of CVDs.

The concept of nano-based platforms represents the basis for multifaceted applications such as diagnosis of pathophysiological conditions of any particular disease and critically modulating the therapeutic measures from a single platform. The nano-based theranostics are regularly used in cancer diagnosis and therapies. The promising theranostic aspects of nano-based cancer therapy platforms gained considerable interest in the last few years for targeted image-guided treatment of other chronic diseases, including CVDs. Spatially designed nano-systems are considered the highly decorated arsenal to treat atherosclerosis [36] (Figure 3). As per the recent trends, FDA-approved nanoparticles such as PLGA, hyaluronic acid, and liposomes gained considerable attention for atherosclerosis treatment. These nanomaterials tend to imitate high-density lipoproteins (HDLs), which are known for anti-atherogenic properties.

Similarly, polymeric nanoparticles and HDL mimicking nanoparticles have also gained the center of attraction for determining the bioimaging and therapeutic modalities in managing atherosclerosis [45]. In the quest for novel nanotherapeutics for diagnosis and treatment of atherosclerosis, a biologically active natural polymer, hyaluronic acid (HA), was exploited for nanoparticle synthesis. The amine-functionalized hyaluronic acid-nanoparticles (HA-NPs) labeled with a fluorescent or radionuclide agent effectively promote macrophages’ logistic uptake and mediate ablation of atherosclerotic plaques. The fluorescent agent presence served as a potential contrasting agent and provided a platform for bioimaging modalities [46]. In the last decade, a significant contribution has been made in the field of designing and developing novel nano-based theranostic agents for the diagnosis and therapy of atherosclerotic plaques through the common platform (Table 1).

Angiogenesis is a standard process during atherosclerosis growth and progression. Hence, anti-angiogenic factors and drug moieties are being considered therapeutic modules for atherosclerosis treatment. For more specificity, the anti-angiogenic drug, perfluorocarbon, was designed into the phospholipid bilayer-based nano-emulsions targeting the α_v_β_3_ integrin and thus served as multimodal nanocomplexes for diagnosis and therapy [43]. In addition, inorganic nanoparticles, magnetic nanoparticles, polymeric nanoparticles, and micelles are also considered promising nanocarriers for target-oriented delivery of a wide range of drug moieties, antibodies and photosensitizers. One of the interesting aspects of considering nanomaterials as theranostic agents for atherosclerotic therapeutics is their tunability to utilize various bioimaging modalities to diagnose disease progression [57]. Since the nano-based platforms provide a wide range of diagnostic imaging and therapeutic applications for atherosclerosis, an array of nano-based carriers are actively exploited for vascular disease management.

The characteristic physicochemical properties such as high SPR, absorption capability, optical properties, biocompatibility and ability to bind to targeting agents provide the functional attributes of gold nanoparticles (AuNPs) as bioimaging and therapeutic modules. The high SPR effect of AuNPs is being deployed for photothermal therapy (PTT), where AuNPs promote macrophage depletion by transducing the light energy into heat in atherosclerotic conditions. Inorganic nanoparticles are being actively employed for PTT utilizing spatial wavelength of near-infrared laser light, leading to intracellular hyperthermia. The use of gold nanorods and silica-gold hybrid nanoparticles was reported to work in tandem with PTT and induce a series of physiological events, including the macrophages apoptosis and reduced risk of cardiovascular mortality by decreasing the total atheroma volume, respectively [58]. AuNPs also act as promising carriers for photosensitizers during photodynamic therapy (PDT). AuNPs facilitate the binding of photosensitizers to the atherosclerotic plaques and mediates the plaques’ depletion by generating reactive oxygen species (ROS) when irradiated. The surface modification and functionalization of AuNPs further improved the therapeutic measures under consideration. The PEGylated aminolevulinic acid-coated AuNPs also exhibited a significant effect on plaque macrophage content [41].

Similarly, mesoporous silica-coated conversion nanoparticles are being exploited as a platform for photosensitizers to trigger NIR-mediated PDT. Under NIR irradiation’s influence, a significant decrease in the intracellular lipids and an increased influx of cholesterol efflux were observed, suggesting an antagonistic effect on atherosclerosis [59,60]. In the early 2000s, a new therapeutic strategy evolved, known as plasmonic PTT (PPTT), which is considered for targeting the atherosclerotic plaque formation by exploiting the infrared properties of noble metal nanoparticles (specifically AuNPs) under the guidance of magnetic resonance [61]. The spatially designed multifunctional AuNPs could bring a paradigm shift in the current therapeutic intervention concept for CVDs, including atherosclerosis. The involvement of AuNPs as contrasting agents for early diagnosis through CT imaging exhibited high-density resolution of the deep tissues under consideration and could be influential in atherosclerosis bioimaging [58].

Paramagnetic nanoparticles are highly effective in the diagnosis and treatment of atherosclerosis. Anti-angiogenic factors could be encapsulated into the magnetically active nanomaterials for effective clearance of atherosclerotic plaques associated with angiogenesis. The optical properties also facilitate the bioimaging module, which further enables therapeutic measures under consideration [42]. Other inorganic nanoparticles, including iron oxide nanoparticles (IONPs), are also being exploited for the management of atherosclerosis. When iron oxide nanoparticles are used combined with conventional bioimaging modalities (i.e., MRI and PET scan), highly sensitive and highly spatial resolution were observed, suggesting their candidature for bioimaging modalities [58]. Since IONPs exhibit superparamagnetic properties (SPIONs), they could be exploited as alternative contrasting agents for conventional diagnostic imaging techniques, including MRI. The ease of synthesis, ease of biofunctionalization and chemical modification are the functional attributes of SPIONs depicting their candidature for bioimaging and therapeutics [62]. SPIONS-based multimodal imaging technique enables atherosclerosis diagnosis as it could be exploited to image the pathophysiological conditions within the macrophages and other biological moieties with high specificity [40]. Besides being used as bioimaging agents, SPIONS also provided platforms for encapsulation of drug moieties of interest, followed by guiding the drug moieties towards the target sites under the influence of magnetic fields [40]. The attributes such as surface functionalization and modification facilitate selective targeting and image-guided therapies. As discussed earlier, macrophages are considered a promising target when designing nanotheranostic platforms to manage atherosclerosis. In this context, cross-linked dextran-coated iron oxide nanoparticles (CLIO-NPs) were prepared in a combination of a chlorine-based photosensitizer, i.e., meso-tetra (*m*-hydroxyphenyl) chlorin (THPC) for macrophage targeted atherosclerosis therapeutics under the influence of near-infrared light. Based upon the promising results, it could be suggested that nanocarriers-based PDT may provide an added advantage to deep tissue imaging and therapy [47,63].

Based upon the characteristic physicochemical properties such as biocompatibility, stability, high drug payload, sustained-release efficacy, localized drug targeting, and relatively low toxicity, the lipid-based nanoparticles, including liposomes, are being actively exploited for diagnosis and therapies of atherosclerosis. Since the liposomal carrier could incorporate both hydrophilic and hydrophobic drug molecules, encapsulation of drug moieties and antibiotics of different specificity are encapsulated into the liposomal carrier for diagnosis and/or therapy for atherosclerosis [57,64]. When combined with an anti-proliferative agent, paclitaxel, earlier, cholesterol-rich nano-emulsions exhibited a promising reduction in the size of the atherosclerotic lesions [65]. The exploration of novel hybrid nanomaterials for diagnosis and therapeutics gained recent recognition as the combination of spatial physicochemical attributes provided new dimensions to the current understanding of therapeutic measures. In this context, synthetic polymer-lipid hybrid nanoparticles were designed for the treatment of atherosclerosis. The spatially designed hybrid nanomaterials specifically target multiple target sites due to the combination of dual-responsive nanomaterials. Besides, the hybrid nanomaterials also allowed encapsulation of high SPR exhibiting inorganic nanoparticles (AuNPs, SPIONs, and QDs) as a potential contrasting agent that facilitates the bioimaging modalities of the highly designed hybrid nanomaterials. Thus, hybrid nanomaterials could provide a new basis for atherosclerosis prognosis and therapeutic management [66]. Earlier, spatially designed solid-lipid nanoparticles (SLN) conjugated with a contrasting agent (iron oxide nanoparticles) and prostacyclin exhibited a reduction in platelet aggregation and thus could be instrumental in atherosclerosis theranostics [56].

Understanding the pathophysiology of atherosclerosis, it was observed that CC chemokine receptor 2 (CCR2) tends to be highly expressed on inflammatory cells and in the atherosclerotic plaques. Hence, targeting the transcriptomic profiling of CCR2 could pave the way for effective therapeutics in atherosclerosis management. In this context, self-assembled specific peptide-based nanoparticles were spatially designed to target CCR2. Since CCR2 is associated with atherosclerosis progression, the high affinity towards CCR2 could provide the platform for both diagnosis and therapeutics of atherosclerosis. The spatially designed peptide-based nanoparticles inhibited the expression of CCR2 by mediating a cascade of events such as ameliorating the monocyte migration and modulating the inflammatory response of IL-1β [67]. It is evident from pathophysiological studies, low-density lipoproteins (LDLs) naturally accumulate in the atherosclerotic plaques and associate with the proteoglycans and remnant ROS. As a result, an increased accumulation of macrophages occurs at the plaque site mediated through a number of receptors such as Toll-like receptors (TLR4), lectin-like receptors (LOX-R) and scavenger receptor A (SRA). As the LDLs primarily involve in atherosclerotic progression, squalene (precursor for cholesterol) was employed as a biomimetic carrier for specific interaction with the LDLs. When the biomimetic carrier is conjugated with specific fluorescent molecules, it could be utilized for bioimaging and image-guided therapy for atherosclerosis [68].

In recent times, considerable interest is being laid upon using naturally derived molecules, mostly from plant sources, for therapeutic applications. Plant-based natural products are also observed for the synthesis of biogenic nanoparticles. Besides, plant-based compounds are also spatially incorporated into specific nanocarriers as effective therapeutics. In this context, plant-based compounds loaded into nanocarriers also gained considerable attention for the treatment of vascular diseases, including CVDs, as it is evident that inorganic nanoparticles are being actively used as contrast agents and therapeutic drug delivery. Recently, curcumin, a bioactive compound, was incorporated into titanium oxide nanoparticles and conjugated with the MCP-1 antibody. The resulting nanoplatforms exhibited promising attributes as contrast agents for MRI and could be utilized for the early diagnosis of atherosclerosis [69].

## 4. Nanotheranostics for Aneurysm

### 4.1. Aneurysm: An Overview

The pathophysiological condition of ‘aneurysm’ is inferred to the enlargement/ bulging/ distention of the artery due to the weakening of the arterial wall and proved to be associated with fatal complications when ruptured at a later stage of development. As per the recent trends and reports from the Centers for Disease Control and Prevention (CDC), an aortic aneurysm causes the death of approximately 25,000 people every year in the United States. The excessive deposition of fats and cholesterol, high blood pressure, obesity, smoking, age, and family history of heart diseases are the common risk factors associated with aneurysms. Based upon the localization of arterial distention, the aneurysm is categorized into the aortic aneurysm (linked to the main aorta carrying blood from left ventricles of the heart through the chest and abdomen), cerebral aneurysm/intracranial aneurysm (arteries supplying blood to the brain) and peripheral aneurysm (occurs at peripheral arteries). The aneurysm associated with the heart is further categorized into a more pronounced abdominal aortic aneurysm (AAA) and lesser-known thoracic aortic aneurysm (TAA). Both aortic and cerebral aneurysms were highly fatal and could lead to lethal conditions due to stroke. Among the different types of aneurysms, AAA gained considerable attention as it is the primary cause of the high mortality rate and morbidity in older adults. When ruptured, the fatality of AAAs could be observed from several pathophysiological events such as neovascularization, increased reactive oxygen species (ROS) generation, apoptosis of vascular smooth muscles, and degradation of aortic lamellae and infiltration of inflammatory cells [70]. The above-mentioned pathophysiological events lead to severe medical complications such as thromboembolism, rupture of the aorta, severe chest and/or back pain, angina (leading to myocardial ischemia and heart attack) and severe headache due to subarachnoid hemorrhage (SAH). Since the occurrence of AAAs is associated with a high prevalence of CVDs, including myocardial infarction, stroke, ischemic heart disease and heart failure, the rate of cardiovascular mortality also increases at an alarming rate when the patients were diagnosed with AAAs [71].

As per the therapeutic procedures, mechanical and invasive intervention proved to be effective in treating aneurysms. The invasive procedures involve either open surgery to fit a synthetic or stent-graft or the endovascular repair using stent-graft surgery. For open-type AAA repair, the abdomen is incised to expose the aorta, followed by applying a graft into the aneurysm site. Meanwhile, in the endovascular method, an endovascular graft is inserted through the incision using a catheter followed by the graft’s repositioning to seal the aneurysm. Open surgery is generally associated with perioperative mortality and morbidity. On the other hand, the endovascular repair mechanism is generally contributing 60–70% success rate as this mechanism is also associated with several complications such as the requirement of reintervention due to reperfusion (bleeding around the graft), blockage of the stent, nerve damage, kidney failure and erectile dysfunction [70]. Thus, it is essential to develop alternative non-invasive therapeutic measures for the treatment of aneurysms.

### 4.2. Nano-Based Platforms for Diagnosis and Treatment of Aneurysm

**Figure 4:**
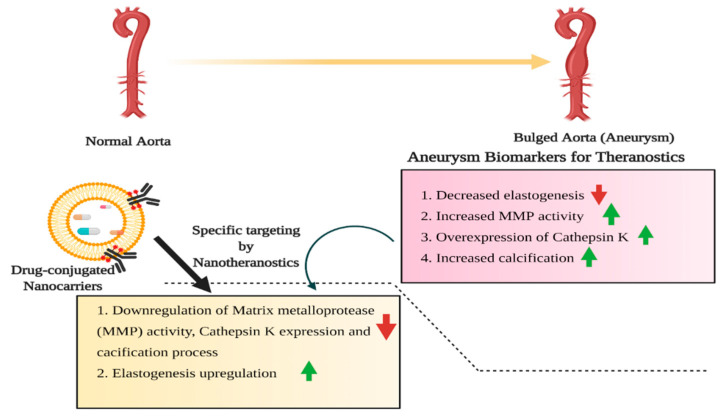
Schematic overview of aortic aneurysms (AA), an important aspect of CVDs and the potential biomarkers considered as a putative target for the diagnosis and therapeutic measures for the management of aneurysms.

Based upon the severity of aneurysms and limitations associated with the conventional invasive procedures, highly sufficient and alternative non-invasive approaches for diagnosis and therapeutics are the need of the hour. As the multifaceted nanoplatforms exhibit promising physicochemical properties, they are being considered effective theranostic agents. The exploitation of nano-based platforms improved the early diagnosis due to the spatial optical and magnetic properties. It provided a basis for encapsulating drug moieties of interest for targeted delivery to manage aneurysms (Figure 4). It is known that AAAs leads to the upregulation of matrix metalloprotease resulting in the degradation of elastin and elastin matrix of the aortic wall and thus associated with progressive loss of elasticity.

Targeting the regulation of matrix metalloprotease and delaying the growth and progression of AAAs are viable methodologies for therapeutics. Though doxycycline (tetracycline-based antibiotic) is clinically reported for slowing the AAAs progression, relative systemic side effects and promoting the inhibition of elastin matrix deposition limit its widespread therapeutic application [72,73]. In this context, surface-functionalized doxycycline-loaded poly (lactic-co-glycolic acid) (PLGA) nanoparticles were designed to overcome the limitations associated with doxycycline. The spatially designed, biocompatible doxycycline loaded PLGA nanoparticles critically enhanced aortic uptake, followed by improved elastin binding. The sustained release of doxycycline from the nanoplatforms progressively down-regulated the matrix metalloprotease activity [72]. The encapsulation of superparamagnetic iron oxide nanoparticles (SPIONs) into the spatially designed doxycycline-loaded PLGA nanoparticles further improved the elastic matrix deposition at the target sites by inhibiting the specific matrix metalloprotease-2 and matrix metalloprotease-9 activities. Thus, incorporating additional and detailed modalities into the designed nanoplatforms could improve therapeutic measures for AAAs [74].

The localized delivery of doxycycline from PLGA based nanomaterials improved the elastogenesis and strongly inhibited the matrix metalloprotease activity [75]. The characteristic therapeutic effect of sustained delivery of doxycycline is achieved through inhibiting the expression and phosphorylation of the regulatory protein, c-Jun-N-terminal kinase 2 (JNK 2) and consequent upregulation of transforming growth factor-beta (TGF-β1) [76]. In a subsequent study, doxycycline loaded into cationic amphiphile modified submicron particles were surface modified with a specific antibody, which acts upon the lysosomal protease, cathepsin K. Since cathepsin K tend to overexpressed during the growth and progression of AAAs, the spatially designed platforms not only controlled the sustained release of doxycycline but also specifically act upon the cathepsin K and thus controls the regulation of its expression profile. Thus, the designed particles achieved bimodal therapeutics by maintaining the elastogenic properties of aortic walls without systemic side effects upon release of doxycycline and, on the other hand exhibiting the anti-proteolytic activity by down-regulating the expression of cathepsin K [73].

For therapeutic intervention in targeting the AAAs, it is equally important to diagnose the growth and pathophysiological changes. Conventional anatomical imaging techniques such as Computed tomography (CT), magnetic resonance imaging (MRI), nuclear imaging and optical imaging are widely used for diagnosis. Similarly, coronary artery aneurysms, coronary angiography, intravascular ultrasound, CT angiography, echocardiography and coronary MR angiography are regularly used for diagnosis [77]. The limitations associated with CT imaging are systemic toxicity (irrational use of iodine and/or gadolinium-based contrasting agents) and the inability to provide exact pathological information about extracellular matrix degradation, which further aggravate the conditions. In this context, optically active, relatively low toxic and exhibiting high absorption co-efficient gold nanoparticles (AuNPs) could be considered as promising alternative contrasting agents for CT imaging. Due to the tunability for surface modification associated with AuNPs, thus improved the sustained drug delivery at the target sites. The polyethylene glycol (PEG) modified AuNPs not only characteristically improved the imaging quality with in-depth analysis of pathophysiological conditions related to AAAs but also critically reduce the use of contrasting agents, thus minimizing the relative toxicity [78]. Since elastin is considered an important biomarker in the progression of aortic aneurysms, spatially designed nanoparticles targeting the elastin protein could facilitate a new dimension to current knowledge of diagnosis and therapy of AAAs. In this context, elastin-targeted AuNPs were formulated as a potential tool for diagnosing aneurysms and thus mediating the appropriate therapeutic approach [79].

For early diagnosis and treatment of aortic aneurysms, lipid-based nanomaterials are considered to be highly effective. These nanomaterials can encapsulate both hydrophilic and hydrophobic drug molecules with a high drug payload. In addition, ease of surface functionalization, controlled release profile, biocompatibility, and localized drug targeting are the most important lipid-based nanoparticles’ attributes. Paramagnetic/fluorescent micellar nanomaterials functionalized with collagen-binding protein (CAN-35) exhibited promising aspects as diagnostic tools to visualize fibrotic response in the aortic wall associated with aneurysms [80]. Recently, FDA-approved statin (pitavastatin) was encapsulated into liposomes and developed a target recognizing liposomal nanocarrier by conjugating the drug-containing liposomes biotinylated bio-nanocapsules. The hybrid nanomaterials enable the binding of the nanocarriers to the targeted graft followed by sustained release of the loaded drug, which significantly down-regulated the matrix metalloprotease expression. Hence, the nanohybrid system could be considered as promising therapeutic machinery for aortic aneurysms [81]. The exciting aspect of selecting nano-based platforms for disease diagnosis and therapy is their ability to accumulate different kinds of drug moieties of interest. It was observed that aortic aneurysms are associated with several pathophysiological events, including vascular calcification and degradation of the elastic lamina. In order to achieve dual-target specific activities, the nanomaterials were designed to conjugate with particular materials such as ethylene diamine tetraacetic acid (EDTA) and pentagalloyl glucose (PGG) for dual-responsive events such as mineral deposition minimization and elastic lamina restoration, respectively. The spatially designed nanomaterials were observed to control macrophages’ accumulation, reduction of matrix metalloprotease activity, vascular calcification, and elastic lamina degeneration [82].

## 5. Nanotheranostics for Myocardial Infarction

### 5.1. Myocardial Infarction: An Overview

Myocardial infarction refers to the obstruction of coronary arteries resulting in prolonged ischemia and loss of cardiomyocytes, vascular cells and interstitial cells [83]. Myocardial infarction or cardiopathy becomes a severe public health issue as acute myocardial infarction is associated with a high mortality rate and morbidity [84]. The increased myocardial infarction incidence results from the imbalance between the oxygen supply to the myocardial tissues concerning the oxygen demand to the myocardial tissue [85,86]. Myocardial infarction is commonly known as a heart attack. A portion of the heart did not receive a fresh supply of oxygen due to a coronary artery blockage. Since the coronary arteries’ function lies in collecting newly oxygenated blood to the myocardium, the deprived supply of oxygen to the myocardium leads to the death of myocardial cells (called infarct). The condition of hypercholesterolemia, abnormally low level of good cholesterol (HDL), high blood pressure, diabetic disease, obesity, and lack of regular physical activity are the risk factors associated with myocardial infarction (heart attack). The symptoms of myocardial infarction include chest pain, irregular heart beating, weakness, fatigue, profuse sweating, breathlessness, nausea, and vomiting [87]. Since the mortality and morbidity rates are comparatively higher in myocardial infarction, early diagnosis and proper therapeutic measures should be undertaken.

### 5.2. Nano-Based Platforms for Diagnosis and Treatment of Myocardial Infarction

**Figure 5:**
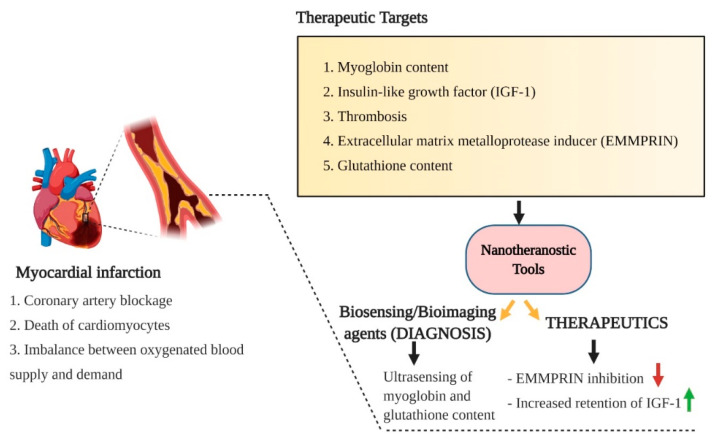
A schematic overview of the pathophysiological risk factors associated with myocardial infarction (heart attack), important healthcare settings in CVDs and the potential pathophysiological targets for early diagnosis and therapy of myocardial infarction using nanotheranostic platforms.

In the quest for novel non-invasive techniques for early diagnosis and treatment of myocardial infarctions, the conventional bioimaging modalities were developed in combination with precise nanomaterials. Nanomaterials’ physicochemical and optical properties are considered as prolific contrasting agents for bioimaging modalities with improved resolution. In this regard, pH-sensitive albumin nanocomposites in conjugation with manganese dioxide (MnO_2_) motifs were engineered. The novel nanocomposites exhibited a multi-fold increase in the relaxation of myocardial infarction. The nanocomposites were spatially designed to accumulate in the heart’s ischemic tissue, from where they were uptake by macrophages. Once the nanocomposites were uptake, Mn^2+^ was released and used as a biomarker for high precision bioimaging [84]. The pathophysiological conditions of myocardial infarction are generally induced by myocardial ischemia and hypoxia, which ultimately trigger ROS production. The spatial over-production of ROS further leads to reduced expression of the glutathione level. Hence, detecting the glutathione level could pave the way for diagnosing ischemic heart disease and myocardial infarction. In this regard, carbon dots based ultrasensitive biosensors were developed to detect glutathione levels, which correlates with the pathophysiology of myocardial infarction. The sensitivity of carbon dots-based biosensors was improved by incorporating silver nanoparticles (AgNPs), mesoporous silica and graphene oxide. Among the conjugated nanomaterials-based biosensors, carbon dots in association with mesoporous silica exhibited a promising ultra-sensing ability. Thus, this concept could facilitate the early detection of myocardial infarction and therefore provide necessary inputs for further therapeutics [88] (Figure 5).

Myoglobin is a crucial cardiac biomarker for early detection and diagnosis of myocardial infarction as it released into the bloodstream once there is an incidence of cardiac muscle injury. Hence, monitoring the myoglobin level could facilitate the early detection and appropriate therapeutic measures for myocardial infarction. In this context, Mn-doped zinc oxide nanoparticles (Mn-ZnO NPs) were spatially designed to detect early signs of acute myocardial infarction by monitoring myoglobin content. As evidenced from results, the engineered nanomaterials observed to detect the electrochemical changes in the level of myoglobin and could be considered as ultrasensitive biosensors [89]. For efficient detection and diagnosis of myocardial infarction, AuNPs served as a promising contrasting agent for CT imaging. The conjugation of collagen adhesion into the engineered AuNPs strictly improved the detection of myocardial scar and ischemic injuries and facilitated proper therapeutic measures to be taken into consideration [90]. In an earlier study, the electrochemiluminescence properties of *N*-(aminobutyl)-*N*-(ethylisoluminol)-functionalized gold nanoparticles (ABEI-AuNPs) and citrate-capped AuNPs were developed for sensitive detection of human cardiac troponin I, an important biomarker for diagnosis of acute myocardial infarction [91,92].

For the detection and monitoring of pathophysiological conditions associated with myocardial tissues, cardiac magnetic resonance imaging (CMR) was developed. Paramagnetic gadolinium (Gd^3+^) is being actively employed as contrasting agents for CMR. As contrasting agents’ role critically influences the detection and monitoring of different pathophysiological conditions, it is essential to the quest for novel nano-based polar agents. In this regard, Gd-based paramagnetic quantum dots in conjugation with specific tripeptide were developed as contrasting agents for detecting and imaging myocardial neovasculature. Though Gadolinium is observed to be safe as a contrasting agent, in few cases, Gd-based contrasting agents’ regular use leads to severe nephrogenic systemic fibrosis. In this context, physiocochemical properties of various nanomaterials physiochemical properties could be considered as alternative and effective contrasting agents for diagnosis purposes. For instance, SPIONs were reported for their ability to detect the difference between normal and infarcted myocardium due to contrasting differential measures [93,94].

Apart from being used as a potential contrasting agent for bioimaging modalities, AuNPs also showed promising therapeutic efficacy. Intravenously administered AuNPs have been shown to critically improve the isoproterenol-induced myocardial infarction by maintaining cardiomyocytes’ architecture and expression of endothelial nitric oxide (eNO) and B-cell lymphoma 2 [95]. In an earlier study, proanthocyanidin-synthesized AuNPs exhibited a characteristic cardioprotective effect by mitigating the isoproterenol-induced myocardial injury [96]. The infiltration of circulating macrophages during myocardial infarction pathophysiological progression is considered a potential biomarker for detecting diseased conditions. The utilization of SPIONs based MRI bioimaging modalities as promising contrasting agents for targeting the infiltrating macrophages to diagnose the pathophysiological responses. The SPIONs based contrasting agents were observed to exhibit higher efficacy and ultra-sensitivity in detecting the macrophages, thus suggesting their importance in the diagnosis and therapy of myocardial infarction [97]. The clinically approved SPIONs (ferucarbotran) for human use exhibited improved visualization of macrophages in infarcted myocardial tissue and thus could be considered as a potent contrasting agent for MRI [98]. The widespread utilization of metal and metal oxide-based inorganic nanoparticles as prolific contrasting agents and drug delivery platforms gained considerable attention in detecting and managing CVDs. In recent times, manganese oxide nanoparticles (MnO NPs) were engineered and directed to use as a contrasting agent for MRI bioimaging modalities for early and sensitive detection of myocardial infarction. The engineered MnO NPs also act as a promising drug delivery vehicle and could be instrumental in treating ischemic heart disease [99].

FDA-approved PLGA-based polymeric nanoparticles are also employed for the treatment of myocardial infarction. For instance, insulin-like growth factor (IGF-1) complexed with PLGA nanoparticles exhibited increased retention of IGF-1 as it is important in regulating myocardial function, growth of cardiomyocytes and cardiomyocytes survival. When IGF-1 was exogenously supplied, when complexed with PLGA nanoparticles, increased cardioprotection incidence, prevention of cardiomyocyte apoptosis, reduction in infarct size, and improved left ventricular incidence ejection fraction (LVEF) were observed [100,101]. The therapeutic efficacy of pitavastatin-loaded PLGA nanoparticles was established in ischemia-reperfusion injury in the myocardium. The engineered nanoparticles loaded with pitavastatin exhibited promising cardioprotective properties by inducing phosphorylation of protein kinase (PI3K)/Akt pathway and inhibiting the inflammation and apoptosis of cardiomyocytes in the myocardium [102]. Lipid-based nanocarriers, especially liposomes, are highly influential in targeted drug delivery with high drug payload and controlled release efficacy. As thrombosis is generally associated with myocardial infarction’s pathophysiology, localized delivery of the thrombolytic drug, urokinase, could be considered to treat myocardial infarction. Liposomal vehicular nanocarriers were designed to encapsulate urokinase and further surface-functionalized with cyclic tripeptide for targeted delivery of urokinase. The functionalization facilitates the selective binding of tripeptide to GPIIb/IIIa receptors present on the activated platelets and allows selective delivery of urokinase at the target sites [103]. The use of stabilizers like polyethylene glycol (PEG) as a surface modification on liposomal nanocarriers further improved the circulation time and prolonged the therapeutic effect in minimizing the infarcted myocardial tissues [104]. Apart from liposomes, paramagnetic micellar nanoparticles functionalized with extracellular matrix metalloproteinase inducer (EMMPRIN) binding peptide, AP-9 (NAP9), also designed to target the EMMPRIN, which plays a pivotal role in inducing matrix metalloprotease activity during ischemic conditions. Thus, targeting EMMPRIN could facilitate the relative repression of the metalloprotease protein expression and thereby regulate the pathophysiology of infarcted myocardium [105]. Lipid-based nanoparticles are considered highly effective not only as diagnostic tools but also as therapeutic drug delivery systems for CVDs. When designed for myocardial infarction management, micellar nanoparticles were observed that were explicitly distributed in the entire infarcted area and thus facilitated the targeted delivery of therapeutic drugs. Meanwhile, liposomal nanocarriers exhibited relatively slower and restricted extravasation suggesting their efficacy in delivering pro-angiogenic drugs. Though both lipid-based nanomaterials exhibited differential functional attributes, they remain the therapeutic platform for myocardial infarction [106].

Cardiac ischemia-induced myocardial infarction is generally associated with elevated ROS and intracellular Ca^2+^ following the reperfusion injury leading to cell death and cardiac hypertrophy. In this regard, spatially designed multifunctional poly(glycidyl methacrylate) (PGMA) nanoparticles loaded with specific peptide (showing affinity towards the alpha-interacting domain (AID) of L-type Ca^2+^ channel) and potent antioxidant molecules (curcumin/resveratrol) for combinatorial therapy. The simultaneous delivery of the specific peptide and antioxidant moieties allowed the reduction in the elevated level of ROS and Ca^2+^ and thus was observed to be involved in minimizing the pathophysiological events associated with ischemic injuries [107].

## 6. Nanotheranostics for Angiogenesis or Regeneration

One of the most exciting aspects of therapies associated with CVDs is the restoration of blood supply, as there are several instances of blood occlusion during the progression of CVDs. The repair of blood vessels is achieved through many inter-related physiological mechanisms such as angiogenesis, vasculogenesis and arteriogenesis. Angiogenesis refers to the restoration of blood vessels by augmenting the host angiogenic response. The process of angiogenesis is a highly regulated mechanism involving the interplay between pro-angiogenic and anti-angiogenic factors. The process of angiogenesis involves a sequential cascade of events, including activation (increased vascular permeability and detachment of pericytes from endothelial cells), progression (degradation of extracellular matrix and migration of endothelial cells), migration, differentiation and maturation [108].

Therapeutic angiogenesis forms the basis for revascularizing the ischemic tissues by promoting specific growth factors and progenitor cells [109]. Gene therapy and stem cell therapy are recognized in therapeutic angiogenesis and employed to improve blood flow, revascularization and myocardial function [110]. In addition to gene and cell-based therapy, protein therapy also received recognition for therapeutic angiogenesis by the direct intervention of angiogenic proteins. The advantages such as tunability in systemic/localized delivery, ease of systemic administration (intravenous and/or intraperitoneal) are associated with protein therapy-based angiogenesis [111]. The vascular endothelial growth factors (VEGFs) mediated therapeutic angiogenesis reported to treat ischemic heart disease [112]. In addition to VEGFs, fibroblast growth factors (FGFs) are also exploited for therapeutic angiogenesis and are administered as recombinant proteins or the genes encoding these proteins [113].

The advent of nano-based delivery systems has revolutionized the healthcare sectors and showed promising aspects in the management of CVDs. Localized targeting, controlled release, and increased circulation time are essential for therapeutic efficacy. In this regard, the factors responsible for angiogenesis also required suitable nano-based delivery systems for promoting angiogenesis. An important factor for angiogenesis is microRNA-126 (miR-126), which plays a crucial role by blocking the expression of sprouty-related EVH1 domain-containing protein 1 (SPRED1). Since SPRED1 is known to inhibit the angiogenic factor, VEGF; blocking the SPRED1 is essential for maintaining the angiogenesis. For effective control of SPRED1, miR-126 loaded PLGA nanoparticles were designed. The nanoparticles allowed the localized and sustained release of miR-126 to inhibit SPRED1 and thus promoted angiogenesis [114]. Based on the importance of pro-angiogenic factors in the process of angiogenesis, deferoxamine-loaded biocompatible PLGA nanoparticles (DFO NPs) were designed and incorporated into the chitosan-hyaluronic acid-based hydrogel. DFO NPs loaded composite hydrogel (mimicking the extracellular matrix) shown to promote neovascularization by sustained delivery of deferoxamine [115]. Earlier, heparin-conjugated PLGA nanoparticles were reported for their role in therapeutic angiogenesis. The biocompatible nanoparticles significantly promote angiogenesis by stimulating the angiogenic growth factors and could be considered to treat ischemic disease [116]. To increase the effect of nano-based delivery platforms on angiogenesis, lipophilic and non-steroidal anti-inflammatory drug, celecoxib was formulated with biocompatible hydrogel-based nano-emulsions. The spatially designed nanomaterials characteristically improved angiogenesis by augmenting the limitations associated with the hydrophobic nature of celecoxib [117]. As angiogenesis is essential for treating ischemia and zinc, it is categorized as a stimulatory effect on angiogenesis; zinc-based metal epigallocatechin-3-gallate (EGCG) nanocapsules were designed for the sustained release of zinc ions to stimulate angiogenesis. The conjugation of EGCG promotes ROS scavenging of inflammatory responses. The engineered metal-based polyphenol nanonetwork exhibited a significant effect on the induction of endothelial cell migration and critically enhanced VEGF’s secretion, thereby facilitating a microenvironment for angiogenesis treatment of ischemic heart disease [118]. Since nano-based platforms with the loaded drug moieties and angiogenic factors facilitate the process of angiogenesis, they could promote revascularization of ischemic heart tissues and repress the progression of tissue infarction [119].

Graphene oxide and reduced graphene oxide specifically induced angiogenesis by modulating the physiological level of ROS, reactive nitrogen species (RNS) and activation of endothelial nitric oxide synthase. PEGylated graphene oxide also promotes angiogenesis with reduced cytotoxic effects [120,121]. Earlier, pro-angiogenic biosynthesized AuNPs were designed to facilitate the process of angiogenesis. Angiogenesis, under the influence of biogenic AuNPs, was achieved through the activation of the Akt pathway and modulating the redox signaling process [122].

## 7. Limitations and Future Perspectives

Several therapeutic drug molecules were developed in the biotechnological and pharmaceutical sectors, with differential functional attributes. For example, few drug moieties can be used as a biomarker for diagnosis purposes, whereas other drug molecules exhibited therapeutic efficacy. Though an array of drug molecules is being employed to manage CVDs, they only temporarily delayed the progress of pathophysiological conditions. Besides, the therapeutic use of these drug molecules also leads to systemic side effects. In this context, nano-based platforms proved to be a dual-responsive system where they could be utilized both as diagnostic and therapeutic tools from a single platform. The localized drug delivery and prolonged therapeutic effect due to increased circulation time also characteristically established the utility of nano-based media [123]. The nanotechnological intervention also facilitates the diagnosis of disease prognosis and relatively associated therapeutic strategies through a common platform. Hence, the nanoplatforms have given a new dimension to design and develop smart nanotheranostic agents for disease diagnosis and therapeutics. Owing to the recent impact on global health issues associated with CVDs, theranostic nanomedicines proved to be effective in complementing conventional diagnosis and therapeutic measures [32]. As evident from earlier studies, nano-based theranostics were found to influence the prognosis of atherosclerosis, angiogenesis and other CVDs by sensitive detection of pathophysiological conditions and also infer appropriate therapeutic measures [124]. The efficacy of nanotheranostics in CVDs lies in the early and sensitive detection of CVDs associated with pathophysiology, controlled and localized drug delivery, and prolonged therapeutic effect [125].

Based upon the differential physicochemical properties, optical characteristics and magnetic behavior, engineered nanomaterials exhibited widespread functional attributes. No doubt, various nanomaterials as theranostics are used for different purposes; their specificity and efficacy are based upon the target specificity. Nanomaterials were designed and engineered for multiple applications with differential advantages and disadvantages. One of the most important limitations of the use of engineered nanomaterials as next-generation nanotheranostic agents is the use of these nanoplatforms is limited to laboratory setup and in the preliminary stage of clinical settings. Hence, it is important to decipher the sustainability and prospective toxicity profile of the engineered nanomaterials before being considered for clinical applications [120]. Though the preclinical studies have established the widespread applications of theranostic nanoplatforms for the efficient management of CVDs, the translational aspect of nanotheranostics to clinical healthcare settings remain unexplored and more intensive and integrative approach needs to be followed with possible toxicity profile and other regulatory issues in mind [126]. In addition, specific targeting of potential biomarkers, innate toxicity of different components associated with naotheranostic platforms, maintenance of the stability of the formulated nanotheranostic agents, production costs and other regulatory issues are associated with the limitations of nanotheranostic agents. Secondly, the targeted therapeutic mechanisms associated with theranostic nanoplatforms differ from person to person and thus, integrative personalized nanomedicines need to be developed for effective diagnosis and therapeutics of several diseases and disorders, including CVDs [127]. Apart from the above-mentioned parameters, ethical issues and cost-effectiveness of large-scale preparation of theranostic nanoplatforms also proved to be a challenging aspect in translating the experimental settings into clinical and market-oriented approaches [64]. Another limiting aspect of nanotheranostics in the maintenance of CVDs and other vascular diseases is that the majority of preclinical studies were reported from small animal models where differential anatomies of vascular diseases are observed as compared to human counterparts. Hence, it is important to validate the efficacy of the engineered nanotheranostic materials in higher animal models for clinical and potential commercial use [35].

## 8. Recent Patents Progress in Cardiovascular Therapeutics

Considerable progress has been made in the last few decades on developing theranostic nanoparticles with differential formulation for multifunctional biomaterials for various biomedical applications and is being patented. This progress could be instrumental in the future for efficient management of CVDs and other diseases [128,129,130]. Similarly, a considerable progress has also been made in the invention of novel biomaterials to manage CVDs. Recently, UV-cross-linkable gelatin methacrylate-based cardiac patch, impregnated with gold nano-rods with improved physicochemical characteristics (high surface area and improved conductivity), was patented (US20170143871 A1). In another recent invention, nanoparticle-based stem cell conjugates were developed and patented (JP5495215 B2) for treatment in the post-infarction period. For efficient detection and treatment of coronary heart disease, spatially designed nanomagnetic particles were developed and patented (CN102085380A). Most recently, Chinese traditional medicine combined with solid lipid nanoparticles was patented (CN103027981B) to treat coronary heart disease efficiently. Though considerable progress has been made in patenting the nanotherapeutics in diagnosing and treating CVDs, their translation into clinical settings remains obscured due to stringent regulatory guidelines in developing nano-based therapeutics [131,132].

Since the engineered nanomaterials with suitable therapeutic drug moieties exhibited certain advantages as well as few limitations, however, the ease of synthesis, surface modification, and functionalization provided new avenues to re-engineer the theranostic nanomaterials of particular interest to improve diagnosis and therapy efficacy. Though the engineered theranostic nanoparticles possess few differential limitations, the advent of nanotheranostics has opened a new door for a new scope and scintillating avenues for developing novel platforms to improve healthcare settings and minimize the nuisances of different pathophysiological conditions. As discussed earlier, the intervention of nanotheranostics has revolutionized our knowledge to tackle the problems associated with CVDs by complementing the available diagnostic and therapeutic tools. The increased incidence of nanotheranostics in biomedical sectors emerged as prolific non-invasive techniques, thereby augmenting the limitations associated with traditional invasive procedures to treat CVDs. No doubt, nanotheranostics constitute widespread applications for disease diagnosis and therapies, but it is also important to regulate these engineered theranostic nanoparticles’ rational use. One more interesting aspect in utilizing theranostic nanoparticles is the imbalance between the number of different theranostic agents reported for their widespread biomedical applications and the number of engineered theranostic nanoparticles that cross the clinical barriers and are employed for human use. In this context, regulatory bodies should evaluate and formulate regulatory guidelines to transform theranostic nanoparticles’ laboratory settings to clinical settings followed by human consumption and/or use.

## 9. Conclusions

Technology intervention and improved healthcare settings have provided a cue to diagnose and develop one or other therapeutic measures for CVDs. In the development of novel therapeutics, the emergence of theranostic nanoparticles allows dual-responsive modalities to diagnose the pathophysiological conditions and provides therapeutic modalities through a single platform. The impact of nanotheranostics in cancer biology has provided a new dimension to design and develop novel nanotheranostics to manage other diseases and disorders. Though the intervention of nanotheranostics in the management of CVDs is a new venture in the healthcare settings, the impact observed has revolutionized our understanding of the pathophysiology of CVDs and interpreted appropriate therapeutic measures. The spatially designed theranostic nanoparticles exhibited promising influence in augmenting atherosclerosis progression, ischemic heart disease, myocardial infarction, and aneurysms. Besides, the theranostic nanoparticles also tend to promote angiogenesis, which is essential for maintaining the cardiovascular apparatus’s health. In the last few years, the incidence of theranostic nanoparticles employed for the management of CVDs was increased exponentially in laboratory settings. It is essential to transform theranostic nanoparticles’ efficacy into clinical health settings before being considered for human use.

## Figures and Tables

**Table 1:** List of notable theranostic nanoparticles designed and developed for the diagnosis and therapy of atherosclerosis, an important factor of cardiovascular diseases (CVDs) since the 2010s.

Sl. No.	Theranostic Agents	Bioimaging Modalities	Therapeutic Modalities	Target	References
1.	Cross-linked dextran-coated iron oxide (CLIO) magnetofluorescent nanoparticles	Fluorescence imaging/MRI	meso-tetra(*m*-hydroxyphenyl)chlorin (THPC)	Macrophagic ablation in atherosclerosis	[47]
2.	Protease-mediated theranostic agent	Near-infrared fluorescence imaging	Cathepsin-B activatable theranostic agent (L-SR15)	Plaque-destabilizing Cathepsin-B activity by selectively eliminating macrophages	[48]
3.	Gold nanorods	Computed tomography (CT) imaging	Photothermally active near-infrared irradiation	Ablation of inflammatory macrophages	[49]
4.	Silica coated plasmonic gold nanorods	Combined intravascular ultrasound (IVUS) and intravascular photoacoustic (IVPA) imaging	Continuous-wave laser	Atherosclerotic plaque management	[50]
5.	Single-walled Carbon nanotubes (SWCNTs)	Near-infrared fluorescence imaging	Photothermally active near-infrared irradiation	Macrophagic apoptosis	[51]
6.	^18^F-fluorodeoxyglucose labeled liposomal glucocorticoid (L-PLP)	Positron emission tomography (PET)/ magnetic resonance imaging (MRI)	Glucocorticoid (PLP)	Management of atherosclerotic lesions	[52]
7.	Doxorubicin-loaded hyaluronic acid-polypyrrole nanoparticles	Fluorescence imaging	Doxorubicin	Targeting proliferating macrophages	[53]
8.	High-density lipoprotein-like magnetic nanostructures	MRI	High-density lipoprotein	Reverse cholesterol transport	[54]
9.	Hybrid lipid–latex (LiLa) nanoparticles	MRI	Rosiglitazone	Targeting proliferating macrophages in atherosclerosis	[55]
10.	Solid-Lipid nanoparticles (SLNs)	MRI	α-tocopherol or prostacyclin (PGI2)	Platelet aggregation	[56]

## Data Availability

No new data were created or analyzed in this study. Data sharing is not applicable to this article.
